# Cellular Models in Schizophrenia Research

**DOI:** 10.3390/ijms22168518

**Published:** 2021-08-07

**Authors:** Dmitrii A. Abashkin, Artemii O. Kurishev, Dmitry S. Karpov, Vera E. Golimbet

**Affiliations:** 1 Mental Health Research Center, Clinical Genetics Laboratory, Kashirskoe Sh. 34, 115522 Moscow, Russia; dimabashkin@gmail.com (D.A.A.); kurishartt@gmail.com (A.O.K.); aleom@yandex.ru (D.S.K.); 2 Center for Precision Genome Editing and Genetic Technologies for Biomedicine, Engelhardt Institute of Molecular Biology, Russian Academy of Sciences, Vavilov Str. 32, 119991 Moscow, Russia

**Keywords:** schizophrenia, model validity, cell lines, multicellular models, CRISPR/Cas9

## Abstract

Schizophrenia (SZ) is a prevalent functional psychosis characterized by clinical behavioural symptoms and underlying abnormalities in brain function. Genome-wide association studies (GWAS) of schizophrenia have revealed many loci that do not directly identify processes disturbed in the disease. For this reason, the development of cellular models containing SZ-associated variations has become a focus in the post-GWAS research era. The application of revolutionary clustered regularly interspaced palindromic repeats CRISPR/Cas9 gene-editing tools, along with recently developed technologies for cultivating brain organoids in vitro, have opened new perspectives for the construction of these models. In general, cellular models are intended to unravel particular biological phenomena. They can provide the missing link between schizophrenia-related phenotypic features (such as transcriptional dysregulation, oxidative stress and synaptic dysregulation) and data from pathomorphological, electrophysiological and behavioural studies. The objectives of this review are the systematization and classification of cellular models of schizophrenia, based on their complexity and validity for understanding schizophrenia-related phenotypes.

## 1. Introduction

Schizophrenia (SZ) is a severe mental disease affecting 1% of the population globally. Three partially overlapping symptom clusters characterize SZ: positive symptoms (e.g., delusions and hallucinations), negative symptoms (e.g., anhedonia, social withdrawal and blunted affect) and cognitive dysfunction. The aetiology and pathogenesis of this disease are largely unknown. There are several hypotheses to explain the origin of SZ. It may result from neurotransmitter misbalances, as suggested in the dopamine, glutamate and gamma-aminobutyric acid (GABA) hypotheses, or from changes in inflammation-related biochemical pathways (the kynurenic acid hypothesis). Both environmental and genetic factors are involved in the pathogenesis of SZ, the latter playing a pivotal role in disease risk; the heritability of SZ is estimated at 70 to 85% [1,2]. The genetic architecture of SZ includes rare gene mutations, copy number variations (CNVs), deletions (e.g., 22q11.2 and 3q29) and chromosome translocations (e.g., disrupted in schizophrenia 1 DISC1) that contribute to the risk of SZ. Genome-wide association studies (GWAS) have significantly deepened our understanding of the genetic architecture of the disease by identifying common genetic variants associated with SZ that reside in both coding and non-coding regions of the genome. A recent meta-analysis of several GWAS identified more than 150 loci associated with SZ [3]. Data from GWAS have rapidly become an essential source of new information on SZ-associated molecular mechanisms. Dysregulation at the epigenetic level involving DNA and histone modifications may also play a role in the aetiology of SZ [4]. Unravelling the molecular mechanisms of the disease is complicated by the highly heterogeneous cellular composition of the brain. Different brain areas may display varying degrees of the abnormalities associated with SZ (pathological changes in myelinated fibres and damage to and degradation of oligodendrocytes) [5].

Appropriate in vitro and in vivo models are needed to elucidate the pathophysiological mechanisms of SZ and develop new drugs to treat it [6]. Cellular models of SZ allow us to reproduce the alterations in the development and function of the brain that underlie the genetic and biochemical aspects of the disease. Besides being a source of fundamental knowledge about SZ, cellular models can serve as platforms for screening potential treatment options.

Here, we describe the most important cellular models used in basic research and drug discovery to investigate the molecular mechanisms associated with SZ at the cellular, biochemical and genetic levels. We categorize the models by their validity and focus on the neuronal cultures and cell types relevant to the pathophysiological mechanisms of SZ. Particular attention is given to CRISPR/Cas9 systems as effective instruments to manipulate the cellular models.

## 2. Constructing a Good Model of SZ

In living SZ patients, brain structure is unique and complex; environmental factors, medication interference and genetic risk are interlinked. Since the human brain is mainly inaccessible except through post-mortem studies when it is altered both intrinsically and by the medications that were taken, researchers need living biological models. Additionally, biological models allow us to establish the causality of SZ through molecular and genetic manipulation.

**Figure 1:**
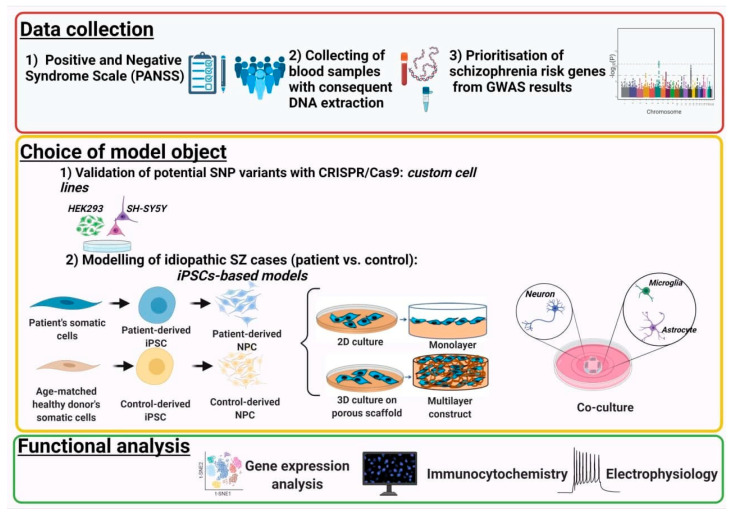
Rational design of SZ models. Data collection: choice of candidate genes and/or patients’ cohorts. Choice of model object: genetically engineered cell models for high-risk SZ cases and autologous iPSC-based models for idiopathic SZ cases. Functional analysis: transcriptome, cellular subtypes identification and morphology analysis and electrophysiology.

A good model of SZ should satisfy the following validity triad: face validity, constructive validity and predictive validity [7]. Face validity for a model of SZ must include the ability to mimic SZ symptoms; these can be observed only in animal models. Cellular SZ models can satisfy the remaining validity dyad. A model of SZ that has constructive validity reproduces disease-related aberrations in the structure and function of the nervous tissue. Predictive validity in an SZ model is the ability to reverse the aberrant phenotype, achieving normality by genetic interventions or drug administration. In general, models can be categorized as those with evolutionarily conserved sequences and human-specific models. The first group includes invertebrates and vertebrates, including non-human primates, and has the advantage of reproducing and investigating evolutionarily conservative high-risk mechanisms associated with SZ. Human-specific models that will be analysed in this review consist of artificial cellular models such as neuroblastoma cell lines, stem cells, induced pluripotent stem cell (iPSC)-derived neurons and multicellular models (neural rosettes, neurospheres and brain organoids). Human-specific models, employing genetically engineered human cells or patient-derived cells, are indispensable to the study of genetic variations in human-specific loci (see Figure 1). Patient-derived cells are used extensively to personalize models of pathogenesis, screen for therapeutics and serve as substrates for brain repair.

### Cell Types Involved in SZ

Although the aetiology of SZ remains unknown, pharmacological and post-mortem studies and in vivo neuroimaging point to neurons and glial cells as key players. Initially, altered neuronal signalling was considered to be the cause of SZ. This hypothesis was supported by the mechanism of antipsychotics, modulating the activity of neurotransmitter receptors, mainly D2 dopamine receptors. However, compounds such as N-methyl-D-aspartate NMDA receptor antagonists can reproduce psychotic symptoms, and glial cells can impact SZ progression. Post-mortem electron microscopy of the dorsolateral prefrontal cortex (DPFC) reveals pathological changes in myelinated fibres and damage and degradation of oligodendrocytes (myelin-producing glial cells). Reduction in the number of dendritic spines in some brain areas and increased protein density in the synapses could be mediated by the action of astroglia, which are involved in the maturation of neurites, particularly of the dendritic spines [8]. Mutations in biosynthetic genes that affect levels of D-serine (the so-called gliotransmitter), which participates in signalling between neuronal and astroglial cells, are known risk factors for SZ. Finally, kynurenic hypotheses of SZ propose pathogenic interactions between astroglia and microglia. Pro-inflammatory cytokines of microglia stimulate kynurenic acid production in astrocytes. Taken together, these observations identify risk-related cellular populations that should be investigated in models of SZ.

## 3. Cellular Models of SZ

Cellular models exhibit constructive and predictive validity. The most popular in vitro model, the SH-SY5Y neuroblastoma cell, is used in neuropsychiatric research. It has the advantage of low cost, ease of culture, reproducibility and available literature [9]. The SH-SY5Y cell line was derived through three successive sub-clones of the parental SK-N-SH cell line. Initially, it was isolated from a metastatic bone tumour biopsy from a 4-year-old female child suffering from neuroblastoma, a rare nerve cell cancer.

### 3.1. Neuroblastoma Model

SH-SY5Y cells display similarities to immature neurons. This cell lineage has dopaminergic and adrenergic properties as well as the proliferative marker (proliferating cell nuclear antigen (PCNA)) and the immature neuronal marker (nestin). Retinoic acid and neurobasal medium induce differentiation of SH-SY5Y cells, including the upregulation of the axonal guidance signalling pathway, growth of neurites and conversion into a neuron-like phenotype. Additionally, the localisation pattern of synapsin-I, a protein associated with the surface of synaptic vesicles, changes during differentiation. This protein shifts from being diffused to aligned with the axonal structure [10].

Both undifferentiated and differentiated SH-SY5Y express the dopamine biosynthesis pathway enzymes and secrete dopamine. The surface of SH-SY5Y contains dopamine, GABA, acetylcholine and glutamate receptors. Taken together, these characteristics confirm the constructive validity of this model [11].

SH-SY5Y cells can be used to reproduce some features of the dopaminergic (DAergic) neurons of the basal ganglia. The main phenotypes with disease-relevant predictive validity for this model are cell survival (resistance to cell damage), membrane potential, the proliferation and migration rates, and the ultrastructure of neurites and synapses.

SH-SY5Y cells treated with the NMDA-antagonist MK-801 were used as a model of impairments in the neurons of patients with SZ. Paliperidone specifically suppressed the action of MK-801. The effect of MK-801 was mediated through the downregulation of the SIRT1/miR-134 signalling pathway and Ca^2+^ influx. Descending glutamatergic neurons send efferent axons to DAergic neurons in the ventral tegmental area (VTA) and substantia nigra (SN). In this model, SH-SY5Y cells represented DAergic neurons of SN or VTA, and MK-801 was proposed to mimic the consequences of prefrontal cortex dysfunction [12]. These data support the predictive validity of the SH-SY5Y cell line as a model.

The convenience of genetic manipulation with SH-SY5Y makes this cell line a valuable object for SZ research. For example, it was used to investigate the molecular mechanisms of the reduction in neurite spines, a well-known vulnerability factor for SZ [13] that has been observed in some regions of post-mortem brains from patients [14]. The shRNA-mediated depletion of transcripts of ULK4, an SZ-associated gene, results in reduced neuritogenesis, neurite branching and cell motility [15]. The observed phenotypes were associated with aberrant remodelling of cytoskeletal components. These observations align with the involvement of Ulk4 in the Wnt pathway (a signalling pathway of cell migration) [16]. Since the developing axons of many neurons receive positional information in the form of extracellular Wnt gradients, dysregulation of the Wnt pathway dosage might play a crucial role in the pathogenesis of mental diseases, SZ among them [17]. The connection between alterations in Ulk4 expression and the regulation of Wnt signalling in SH-SY5Y as a constructive model may contribute to the study of signalling in the neuroplasticity pathways implicated in the aetiology of SZ.

Silencing of the SZ susceptibility gene DISC1 (Disrupted-In-Schizophrenia) by lentiviral infection of shRNA in SH-SY5Y induces mitochondrial fragmentation and defects in respiration. Previously, Western blotting and co-immunoprecipitation data (CoIP) were used to demonstrate that reciprocal chromosomal translocation results in truncated forms of DISC1, such as ∆597-854, which fail to incorporate into the mitochondrial contact site and cristae organizing system (MICOS) protein complex. Fluorescent microscopy showed that the DISC1-depleted cells failed to maintain the mitochondrial oxidative phosphorylation (OXPHOS) complex function [18]. All neural cell subtypes are likely affected by DISC1 truncation because it is expressed during neurodevelopment and regulates the migration and proliferation of neural progenitors [19,20]. DISC1-depleted cells exhibit abnormal mitochondrial morphologies and, as a model with constructive validity, may shed light on the relationship between mitochondrial function and the pathogenesis of SZ.

Similar research was conducted for important SZ candidate genes encoding arsenite methyltransferase (AS3MT). AS3MT is the most statistically significant locus outside the major histocompatibility complex (MHC) region associated with SZ [21]. A human-specific isoform (AS3MTd2d3) lacks AS3MT activity and is more abundant in SZ individuals than in controls [22]. A genome-wide mQTL analysis revealed that AS3MTd2d3 is overrepresented in the brains of SZ patients. To dissect the functional significance of AS3MT domains, a robust protocol for the creation of AS3MT deletion mutants in the SH-SY5Y cell line has been developed.

The forkhead box protein P2 FOXP2 gene encodes a forkhead transcription factor. Mutations in FOXP2 are known to cause developmental verbal dyspraxia, a speech and language disorder. Some polymorphisms of this gene are also associated with SZ. Complex rearrangement involving one copy of chromosomes 7 and 11 might affect some regulatory regions of FOXP2. Notably, the breakpoint in the 7q31.1 chromosome is located 205.5 kb downstream from the 3′ end of FOXP2. Using a luciferase assay, two enhancer regions were identified: the first is located downstream of FOXP2, and the second was found in the intergenic region between the FOXP2 and MDFIC genes. A related neuroblastoma cell line, SK-N-MS, was used to investigate the regulatory role of the two enhancers by targeted deletion using the CRISPR/Cas9 system. The deletion of either enhancer significantly altered the expression of FOXP2 and changed the transcription of six well-known FOXP2 target genes. Thus, identified enhancers regulate the activity of a FOXP2-specific regulatory network in human cell lines. [23]

SHANK2 encodes a protein that functions as a postsynaptic scaffolding protein at glutamatergic synapses in the brain. This protein is essential for synapse formation, development and plasticity [24], and its variants have been linked to the pathogenesis of SZ [25]. The CRISPR/Cas9 system has been used to introduce mono- or bi-allelic SHANK2 frameshift mutations into SH-SY5Y cells. These mutations impair early neuronal differentiation in SH-SY5Y cells, change cell growth properties and reduce pre- and postsynaptic protein expression [26]. These data support the constructive validity of the SH-SY5Y cell line. This cell line and its CRISPR-engineered derivatives may serve as valuable primary screening platforms for antipsychotic drugs [27,28,29]. A neurite outgrowth assay was applied to SH-SY5Y cells to investigate the effect of antipsychotics such as amisulpride and haloperidol on the regulation of neuronal morphology [30]. The results of this experiment supported the predictive validity of this model for drug screening.

Significantly, the tumour origin of SH-SY5Y does not hamper the investigation of neuronal phenotypes in the context of neurodevelopmental and neurodegenerative diseases. However, specific genomic alterations limiting the use of the model should be taken into account. The SH-SY5Y genome shows trisomy of chromosome 7, gain of the short arm of chromosome 2 (2p25.23-2p16.3) and multiple translocations [31].

The findings made using this cellular model and their reproducibility in other disease-relevant models have proved its constructive and predictive validity.

### 3.2. Induced Pluripotent Stem Cells (iPSCs) and iPSC-Derived Neural Cell Models

Since animal models cannot reproduce complex genetic disorders such as SZ and autism-spectrum disorders (ASDs), there is a need for patient-specific modelling tools. iPSCs derived from patients recapitulate each donor’s genotype. Initially, iPSCs were used to model diseases with highly penetrant genetic variants of significant phenotypic effect. However, their application has expanded to modelling psychiatric disorders and generating patient-specific organoids. The ability of iPSC-derived neurons to recapitulate fundamental neuronal functions, including firing action potentials and releasing neurotransmitters, led to the development of functional analysis of SZ-associated genetic variants.

iPSCs are an indispensable source of a plethora of neural lineage cells. The first iPSC cells were generated by the retroviral delivery of Yamanaka transcription factors (Oct4, Sox2, cMyc, Klf4) into somatic cells [32]. Then, techniques for the conversion of adipocytes, fibroblasts, haematopoietic cells and peripheral blood mononuclear cells (PBMCs) to iPSCs were developed [33,34].

iPSCs themselves rarely serve as models of processes associated with SZ. If genetic variations significantly affect the iPSC population, they are probably highly penetrant and responsible for a relatively low proportion of SZ cases. For example, the 22q11.2 deletion was shown to decrease the proliferation rate of iPSCs in neurosphere cultures [35].

The question of the constructive validity of iPSCs and iPSC-derived cells due to intra-subject variability has been discussed extensively [36]. Minimizing intra-individual variability is achieved by choosing the most suitable protocol and following it carefully. Inter-individual variability is reduced by selecting study participants carefully, usually from the same SZ cohorts. Patients with high genetic risk can be compared to healthy controls with the same genetic risk [37].

The drawback of iPSCs is that mutations and CNVs accumulate in somatic cells undergoing reprogramming. Hence, biological repeats are needed in iPSC experiments. iPSCs and iPSC-derived models can recapitulate events taking place in neurodevelopment and are unaffected by secondary events. iPSCs have advantages over post-mortem cells. A 3D neuronal and glial co-culture derived from CRISPR-edited iPSCs at rs4702 (FURIN) exhibited significant transcriptomic and cellular effects, whereas post-mortem data were inconsistent [38]. Late SZ in patients at low genetic risk is likely to have a significant epigenetic impact, which occurs during reprogramming. Notably, gene editing of autologous iPSCs can help assess the penetrance of particular variations on a patient’s genetic background. Edited patient-derived iPSCs can be compared to those of unaffected controls, proving the role of candidate variations. In addition, CRISPR/Cas9 screening of patient-derived iPSCs can be used to identify genetic variations that GWAS studies are underpowered to recognize. It is accepted that idiopathic SZ cases are based on common variations. Through CRISPR/Cas9 screening, iPSCs could be used as a tool to reveal the mechanisms that are disrupted in patients with idiopathic SZ.

Although the reproducibility of iPSC models has been questioned, the appropriate choice of iPSC cells for reprogramming, use of a sufficient number of biological repeats and careful selection of subjects and controls can help overcome their weaknesses.

#### 3.2.1. Neural Progenitor Cells

NPCs are first formed during the iPSC-to-neuron transition. NPCs may also be derived from neural stem cells (NSCs) isolated from the olfactory epithelium (OE) or through direct reprogramming (trans-differentiation) of non-neural cells [39]. NPCs form a homogeneous population after a few passages and continue to proliferate in the presence of fibroblast growth factor 2 as adherent monolayers. In contrast with iPSCs, NPCs tend to be characterized by increased proliferation and apoptosis [40].

After induction with N2 and B27 supplements, with or without the addition of dual-SMAD inhibitors, NPCs organize in neural rosettes. Rosettes are plane structures reminiscent of cross-sections of the neural tube. If clones of NPCs from rosettes are maintained, they differentiate into various types of neurons. Noggin Shh, FGF8 or small-molecule inhibitors can induce migration of the NPCs to specific brain areas, depending on the regulatory pathways active during neurodevelopment [41].

Research performed using NSCs and NPCs has helped to extend the understanding of SZ, so that it is no longer seen as a “synaptic disease” [42] but rather a much more complex neurodevelopmental illness [43]. An imbalance in the proliferation and differentiation of NPCs and a decrease in their migration rate can lead to the thinning and mixing of layers of the cortex that is observed in some SZ patients [44]. A wide range of genetic factors, either highly or lowly penetrant (cumulative), can produce a similar outcome, resulting in excessive cellular stress accompanied by impairment of the proliferation rate, migration and differentiation potential of NPCs. This has been observed in autologous NPCs derived from SZ patients [45] as well as CRISPR/Cas9-engineered NPCs with a depleted main isoform of potassium channel tetramerization domen containing 13 KCTD13 [46].

DISC1 interruption by a balanced Chr(1;11) translocation was associated with SZ in a Scottish family [47]. The mutation was reconstructed in iPSCs, and CRISPR/Cas9 and TALEN technologies were employed to investigate the underlying molecular mechanism [48]. The mutant iPSCs were then differentiated into NPCs and neurons. The mutation led to decreased expression of the longer DISC1 transcript due to nonsense-mediated decay. Transcriptome analysis of the mutant NPCs showed reduced expression of Tbr2 and FOXG1 cell fate markers and supported a subtle dorsal shift in cell fate. Moreover, the results indicate a differential expression of the genes involved in neural development, mental disorders, SZ, Wnt signalling and cell adhesion. A luciferase reporter supported activated Wnt signalling. Wnt antagonists reversed the observed transcriptional signatures [48].

Thus, the NPC model may link mutations to abnormal brain development in schizophrenia.

#### 3.2.2. Glutamatergic Neurons

The glutamate hypothesis explains SZ as a lack of glutamate signalling, mainly via NMDA glutamate receptors. The administration of NMDA receptor non-competitive inhibitors reproduces both positive and negative symptoms of SZ [49]. Glutamatergic neurons can be differentiated from NPCs. The standard protocol leads to a mixed population of neurons, with forebrain glutamatergic neurons being the most prevalent (representing 70 to 90% of neurons) and GABAergic neurons being less numerous [50].

A pathogenic SNP—a 4bp-deletion frameshift in DISC1—is known to be associated with SZ and other mental disorders [51]. The mutation decreases the DISC1 protein level in iPSC-derived glutamatergic neurons, resulting in decreased presynaptic markers and neurotransmitter release defects [52]. Transcriptome studies of mutant glutamatergic neurons have revealed global changes, including presynaptic genes. The phenotype of the mutant cells could be reversed by TALEN-mediated restoration of the DISC1 sequence. The healthy control cells acquired pathogenic phenotypes after introducing the SNP into the wild-type DISC1 sequence. This study established the causality of the DISC1 SNP and demonstrated the contribution of synaptic defects into SZ.

MicroRNA profiling of glutamatergic neurons with 22q11.2 microdeletions, particularly the deletion of DiGeorge syndrome critical region 8 DGCR8, resulted in the dysregulation of a set of miRNAs that control the expression of genes involved in neurotransmitter function, synaptogenesis and neuronal differentiation [53].

A study of the heterozygous 22q11.2 deletion showed abnormal expression of genes involved in apoptosis, mitogen-activated protein kinase MAPK signalling and cell cycle. At the network level, 22q11.2 genes and their co-expressed targets likely play two distinct roles during brain development, with a CDC45-mediated cell cycle pathway involved in embryonic brain development and a PRODH-modulated subnetwork contributing to adolescent brain functions. A possible spatial interaction between the 22q11.2 regions and the human leukocyte antigen 6p21 locus was predicted [54].

Cell type-specific differences in the number of splice variants were identified in the patient’s iPSC-derived glutamatergic neurons and oligodendrocytes with the heterozygous deletion of CNTNAP2. Cells from the proband were compared to those from the unaffected parent with the same mutation. This work demonstrates the level of complexity involved when a genetic variation shows incomplete penetrance, probably due to interactions with an individual’s protective and risk-bearing alleles [37].

Neurons also appear to be indispensable for studying variations in response to pharmacological treatment. This could lead to an understanding of treatment-resistant SZ. A gene expression study of iPSC-derived cortical excitatory glutamatergic neurons of monozygotic twins with clozapine-responsive and -resistant SZ revealed that the cell adhesion genes were expressed differently upon treatment. Proteins of these genes, including protocadherins (PCDHGC3, PCDHA7, etc.) and cadherins (CDH8 (an ASD-risk gene) and DSC3), participate in synapse formation. Further research may shed light on the reasons for treatment-resistant SZ [55]. Specific differentiation conditions can result in the formation of more specialised neuronal subtypes. Upon prolonged incubation with noggin, iPSCs were efficiently converted to a population of neural progenitors that mostly corresponded to a cortical identity. The majority of the neurons were VGLUT1/2-positive and displayed a unipolar and pyramidal morphology index (PMI) corresponding to cortical glutamatergic pyramidal neurons. Neurons from different layers were generated at distinct time points. The earliest-born neurons generated in the cortex are pioneer neurons, followed by deep cortical layers VI and V, upper layers IV, and layers II/III [56]. Pyramidal glutamatergic neurons CA3 and dentate gyrus (DG) neurons of the hippocampus were obtained from iPSCs from patients with SZ and healthy controls. A decrease in the number of spontaneous spike and network bursts in SZ-derived DG-CA3 co-cultures was observed [57].

#### 3.2.3. Dopaminergic (DAergic) Neurons

The hypothesis of dopaminergic system dysfunction is central to SZ and has been one of the most enduring ways of conceptualizing the illness [58]. This hypothesis is supported by studies in animal models and human subjects, including post-mortem and pharmacological studies as well as brain imaging [59]. DAergic neurons can be obtained by the differentiation of iPSCs or direct transformation of fibroblasts [60,61]. However, these protocols may lead to the development of a mixed population of DAergic and non-DAergic cells. Moreover, impaired differentiation of SZ iPSCs into DAergic neurons has been reported [62]. Difficulties with differentiating DAergic neurons from iPSCs may explain their rare usage in molecular studies of genetic variants of SZ. This limitation could be overcome by using a special CRISPR/Cas9-engineered human iPSC line. This cell line expresses tyrosine hydroxylase, a key enzyme of dopamine biosynthesis, C-terminally labelled with mOrange. Thus, correctly differentiated DAergic neurons could be isolated based on fluorescent sorting techniques [63].

iPSC-derived DAergic neurons help to elucidate potential molecular mechanisms linking genetic variants of SZ patients with DAergic neuronal dysfunction. In patients with schizophrenia, Kushima et al. found a rare CNV in the gene of reelin (RELN) that controls neuronal migration in the developing brain [64]. RELN mutations were introduced into allogenic iPSCs using the CRISPR/Cas9 system, and mutated iPSCs were differentiated into homogeneous DAergic neurons [65]. Heterozygous mutation decreased, and homozygous mutation abolished the RELN expression. This in turn led to reduced phosphorylation of the disabled-1 protein, a signal that is required for normal neurodevelopment. Transcriptomic analysis showed upregulation of cell cycle genes and downregulation of genes involved in neuron movement. Consistently, the mutant DAergic neurons lost directionality in the migration test. DAergic neurons differentiated from the patient-derived iPSC or peripheral blood monocytes also show impaired cell movement. Thus, model DAergic neurons allow deciphering molecular and cellular mechanisms that contribute to impaired brain development in patients with SZ.

GWAS studies found that copy number variation at 16p11.2 is strongly associated with SZ [66]. To elucidate molecular mechanisms linking this CNV to SZ, DAergic neurons were derived from iPSCs with a 16p11.2 deletion (16pdel) or duplication (16pdup) [67]. Transcriptome analysis revealed and qRT-PCR validated the overexpression of genes involved in cell migration and proliferation, cytoplasmic vesicle formation and secretion, synapsis, etc. The 16pdel DAergic neurons overexpress synaptic markers and show an increased frequency of excitatory postsynaptic currents in patch-clamp recordings. Patch-clamp experiments also show increased spontaneous activity and excitability of 16pdel DAergic neurons. Consistently, 16pdel DAergic neuronal networks show increased activity and burst firing. Notably, elevated release of dopamine and other catecholamines in SZ DAergic neurons [68] has been shown. Moreover, hyperactivity and burst firing of the DAergic system has been described in SZ patients [69]. The 16p11.2 locus contains KCTD13, a gene encoding regulator of the development of cortical neurons. KCTD13 exerts its function by regulation of the RhoA kinase level via the Cullin-3-ubiquitin ligase complex [70]. Accordingly, reduced KCTD13 expression in 16pdel DAergic neurons leads to RhoA stabilization [67]. Long-term treatment of 16pdel DAergic neurons with the specific RhoA inhibitor rhosin significantly decreases activity and burst firing of the DAergic neuronal network.

These data support the contention that DAergic neurons derived from CRISPR/Cas9-engineered iPSC have both constructive and predictive validity. They could serve as a valuable model of SZ to investigate the pathogenesis of DAergic system-linked GWAS-identified genetic variants.

#### 3.2.4. GABAergic Neurons

Ketamine administration results in acute psychosis, which is accepted as a behavioural model of psychosis in SZ. Since ketamine is an NMDA antagonist, insufficient NMDA receptor input on GABAergic neurons is involved [70]. Genetic studies support these observations of the role of GABAergic neurons. SNPs in the 5′ upstream region of the GAD1 gene, encoding glutamic acid decarboxylase, are associated with SZ [71]. Furthermore, autologous cortical GABAergic interneurons from iPSCs of patients with SZ exhibit downregulation of crucial GABA pathway genes such as glutamate decarboxylase GAD1. Additionally, a deficit of synaptic proteins, including synaptotagmin 1 and neuroligin 2 (NLGN2) (proteins that maintain synaptic connectivity) was observed. Synaptic deficits were ameliorated by the antioxidant N-acetyl-cysteine or overexpression of NLGN2 [72].

Medium spiny neurons, located in basal ganglia, form another subpopulation of GABAergic interneurons. Medium spiny neurons contain D1-/D2- receptors. Recently, a protocol for the differentiation of iPSCs into medium spiny neurons was established [73]. Though most research on iPSC-derived GABAergic neurons addresses Huntington’s disease, there is space for the investigation of their role in SZ.

#### 3.2.5. Oligodendrocytes

The role of glial cells in SZ pathogenesis was mentioned previously. Oligodendrocytes produce myelin, which wraps neuronal axons. A wide range of ultrastructural changes observed in post-mortem oligodendrocytes appeared to be associated with cellular stress, reduced protein and ATP synthesis [74].

iPSCs from SZ patients show a two-time fold reduction in the differentiation rate of O4-positive oligodendrocytes [75]. Studies also show a modest correlation between the percentage of white matter in the brain and that of oligodendrocytes in culture [75].

Interestingly, typical antipsychotics (haloperidol) are shown to stimulate the proliferation of OLs but inhibit maturation. Atypical antipsychotics (clozapine, quetiapine) have the opposite effect. This may explain the better activity of the atypicals against negative symptoms [76]. The potential of therapy targeting oligodendrocytes has been described extensively [77].

### 3.3. Neuronal Cell Models Obtained by Trans-Differentiation of Non-Neural Cells

Patient-specific induced neurons could be obtained directly from fibroblasts by the overexpression of several transcription factors (at least three) together with the addition of miRNA [78]. This approach could be used to obtain functional glutamatergic and GABAergic neurons that can form synapses. Unfortunately, though direct fibroblast reprogramming offers new possibilities for disease modelling, no reports have examined this approach in SZ. The possible reasons could be the low yield of neurons (about 10%), resulting in the need for cell enrichment (however, neurons are tough cells to sort) and the relatively high genetic heterogeneity of neuron populations due to somatic mutations in the fibroblasts.

### 3.4. Non-Neural Lineage Cells

Non-neural cells such as HEK293 are also useful for the investigation of particular genes and proteins linked to SZ. The mutant isoform Kv11.1-3.1 of the primate-specific brain voltage-gated potassium channel lacks a channel-stabilizing Per-Ant-Sim (PAS) domain. This isoform displays abnormal folding and traffic to the plasma membrane and could be responsible for the extended train of action potential in the neurons of patients with SZ. The HEK293 line was used to develop a thallium flux assay to screen drugs that could restore Kv11.1-3.1 function. Proteasome inhibitors restored the traffic of the Kv11.1-3.1 isoform [79].

DPYSL2 is also of interest. It encodes collapsin response mediator protein 2 (CRMP2), which is highly expressed in the central nervous system. CRMP2 is a synaptic protein that controls synaptic transmission via interaction with receptors and channels [80]. SNPs in DPYSL2 are associated with SZ risk [81]. The 5′-untranslated region (5′-UTR) of the DPYSL2B isoform contains polymorphic CT di-nucleotide repeats (DNR). The 13DNR risk allele leads to reduced translation of DPYSL2B in the HEK293 line, as has been shown using the dual-luciferase reporter and ribosome profiling [82]. Two isogenic HEK293 clones containing normal 11DNR and 13DNR risk alleles in 5′-UTR of DPYSL2B were created using the CRISPR/Cas9 system [83]. The 13DNR allele led to the repression of the corresponding CRMP2 isoform. The weak interaction with mTOR effectors mediated the translational repression. Moreover, the 13DNR HEK293 cells had much shorter projections than the 11DNR cells. Interestingly, the transcriptome analysis of 11DNR and 13DNR clones showed differential expression of genes involved in SZ (e.g., ZNF804A). Finally, a connectivity map analysis showed that the transcriptional signatures of the 11DNR/13DNR variants were the opposite of those of the antipsychotics thioridazine, trifluoperazine and prochlorperazine.

These results suggest that HEK293 possesses predictive validity at the biochemical level while being constructively unrelated to neural cells.

## 4. Multicellular and Brain Organoid Models

The pathologies of cortical development in SZ can be investigated in multicellular models, including neural rosettes, neurospheres and brain organoids. Neurospheres and neural rosettes, considered intermediates between iPSCs/NSCs and neurons, also contain valuable information about events in early ontogenesis.

### 4.1. Neural Rosettes

Rosettes possess a basal–apical polarity, nuclear interkinetic motion during mitosis and a lumen. The formation of neural rosettes reproduces events that take place during secondary neurulation in vivo. Rosette polarity and connectivity (the localization and density of cellular contacts) can be studied in SZ cases compared to controls. Defects in complement signalling are known to be associated with SZ. The study of the role of the complement protein C5a and its receptor C5aR1 in neural rosettes has shown that C5a stimulates rosette formation through the PKCζ pathway. iPSC-derived neural rosettes from SZ patients displayed perturbed apical–basal polarity and disrupted adherens junctions relative to controls [84].

### 4.2. Neurospheres

Reynolds and Weiss, in their pioneering work, discovered that isolated NSCs could form floating spherical cultures that they termed “neurospheres”. Neurospheres could also be obtained from iPSCs [85]. The application of the neurosphere model is similar to that of neural rosettes. After 20 days of cultivation, NSCs in neurospheres differentiate into neurons. The addition of EGF/FGF-2 can maintain the proliferative potential of NSCs, and then differentiation is started by the depletion of EGF/FGF-2. NSCs can be obtained from the human olfactory epithelium [86,87,88].

Neurospheres are a good model in which to study the molecular mechanisms of neurodevelopmental abnormalities. Neurospheres from patients with 22q.12 showed reduced size, neural differentiation efficiency, neurite outgrowth and cellular migration distances compared with healthy controls [89]. Moreover, neurospheres from patients with SZ had a decreased neurogenic-to-gliogenic competence ratio. Furthermore, miRNA profiling showed repression of the miRNAs belonging to the miR-17/92 cluster and miR-106a/b that are suppressors of p38alpha expression. The accumulation of p38alpha, promoting the formation of glial cells from precursors at the expense of neurons, led to a 10% decrease in the fraction of neurons in the 22q.12 deletion-neurospheres, and a 10% increase in the fraction of gliocytes. The imbalance in differentiation and reduced migration rates are thought to contribute to the cortical thinning and gliosis observed in patients with 22q.12 [89].

### 4.3. Co-Cultures

Co-cultures help in the investigation of pathogenic mechanisms of intercellular relationships. iPSC-derived glutamatergic pyramidal cortex neurons and GABAergic interneurons from patients with SZ and healthy controls were studied in co-cultures. Comparison of combinations of excitatory and inhibitory neurons showed that an inherent deficiency in inhibitory interneurons underlaid the decrease in synapse formation in patients with SZ compared with controls [72]. Increased synaptic pruning was observed in co-cultures of microgliocytes and extracted synaptic nerve terminals (synaptosomes) obtained from patients with SZ. This methodology required the growth of a mixed population of neurons, which formed synapses. Then, the synaptosomes were extracted through homogenization-fractionation and labelled with a phagolysosome-specific fluorescent dye. The increase in phagocytic uptake indicated synaptic pruning [90].

Since glial cells are critical players in synaptic plasticity in the brain, co-cultures of neurons and glia have deepened our understanding of pathogenic intercellular interactions in SZ.

### 4.4. Brain (Cerebral) Organoids

Organoids are 3D models that reproduce the architecture of brain tissue. They can be used to reproduce the structural changes found in the brains of individuals with SZ. Organoids are derived from iPSCs that are differentiated into neuroepithelium. Most protocols require prolonged incubations in bioreactors with agitation. The stepwise process requires media containing different growth factors and small molecules at each step. However, organoid formation is only partially guided by growth factors. The main factor is self-organization which is controlled by intrinsic stimuli. Recently, methods to grow region-specific organoids—forebrain, midbrain and hypothalamic—have been established. The transcription of genes in human brain organoids at different growth times correlates with their transcription in embryos between 8 and 16 weeks after conception. Brain organoid epigenetics (CpG and non-CpG methylation) strongly correlate with the foetal pattern, and the stages of organoid growth and differentiation are comparable to those in the foetal brain. Since most of the SZ-associated genetic variants lie in non-coding regulatory regions [91], the preservation of epigenetic architecture makes organoids an attractive model to study the epigenetics of SZ. However, single-nucleus RNA sequencing has shown that some cellular populations differ between the foetal brain and brain organoid, and organoids contain a novel cellular population that is not present in the foetal cortex [92,93]. Another problem in this field is reproducibility, although that difficulty was recently overcome in a study of 21 organoids. Of the organoids, 95% generated a virtually indistinguishable compendium of cell types that followed similar developmental trajectories, and the degree of organoid-to-organoid variability was comparable to that of individual endogenous brains [94].

The advantage of organoids is revealed in studying the impact of the FGFR1 signalling network on cortical development during ontogenesis. The consequences of FGFR1 encoding on receptor tyrosine kinase misregulation were studied in idiopathic organoids derived from patients with SZ. The FGFR1 misregulation led to abnormal migration of the Ki67+ NPCs. They scattered from the ventricle zone into the intermediate and cortical zones. The number of TBR1 pioneer neurons and RELN was significantly decreased. Neurons were depleted from the cortex and formed in subcortical regions instead. Experiments with normal organoids overexpressing the dominant-negative or constitutively active mutant FGFR1 or downregulating it with a synthetic inhibitor, PD173074, reproduced the observations made with the SZ organoids. Since the FGFR1 signalling network affects the expression of 84.4% of SZ-associated genes, it would be hard to estimate the physiological effect of FGFR1 misregulation without the constructive validity of the brain organoids [95].

Brain organoids also helped to partially explain the molecular mechanism of SZ pathogenesis associated with the 16p13.11 chromosomal microduplication. This locus contains the gene NDE1 (nuclear distribution protein nudE) and miR-484. Brain organoids from three patients with SZ and five healthy controls, all having the same microduplication, were grown. The SZ organoids showed a markedly reduced number of proliferation zones and asymmetric cell division in those zones. Proliferating cells produced one NPC and one differentiated neuron instead of two NPCs. The inhibition of the NFkB-p65 pathway was the cause of the abnormal proliferation. Stimulating the NFkB pathway with the small-molecule activators SRI-22772 and SRI-22782 or the overexpression of p65 corrected the cell proliferation [96]. These data suggest that brain organoids also have predictive validity.

DISC1 dysfunction, which has been studied extensively, was also recapitulated in organoids. Electroporation of brain organoids with a dominant-negative GFP-DISC1 765-835 form blocks the interaction of NdeI/NdelI with the wild-type DISC1. Since NdeI/NdelI interacts with kinetochore, its impaired interaction with DISC1 leads to a delay in mitosis in radial glial cells. The number of Ki67+ cells in the ventricular zone is significantly reduced compared to cells in the G0 phase. The observed phenotype reproduces the phenotype of organoids from patients with SZ carrying the DISC1 d598-854 deletion or frameshift mutation I808 [97].

While only a few SZ studies have used brain organoids, much work has been done on other mental disorders, including ASDs and bipolar disorder (BD). In some cases, ASD- and BD-associated mechanisms can be translated to SZ. For example, transcriptome analysis of autologous organoids from patients with ASDs shows overexpression of the FOXG-1 transcription factor. This explains the elevated ratio of GABAergic inhibitory neurons to excitatory glutamatergic neurons. At the same time, the populations of Ki67+ proliferating cells were not significantly changed. The RNA interference of FOXG-1 abrogates the overproduction of GABAergic neurons and restores the balance between the number of inhibitory and excitatory neurons [98]. FOXG-1 is also described as an SZ risk gene [99]. The excitatory and inhibitory imbalance is observed in SZ and, as suggested, has a common mechanism with that seen in ASDs [100]. An SZ-associated polymorphism, rs1191551, which was identified in GWAS, was found to interact with FOXG-1 physically. This SNP resides in a putative enhancer, controlling FOXG-1 expression during human cortical development. CRISPR/Cas9-mediated deletion of the site containing the SNP decreases FOXG-1 expression [99]. Taken together, these results suggest that FOXG-1 overexpression may also contribute to SZ by disbalancing the number of inhibitory and excitatory neurons.

The chromodomain-helicase-DNA-binding protein 8 (CHD8) represents another protein that is associated with the pathogenesis of ASDs and SZ [101]. To study a potential mechanism of CHD8′s contribution to ASDs and SZ, CHD8+/- heterozygous organoids were obtained using the CRISPR/Cas9 system, and their transcriptome was analysed [102]. The results suggest that CHD8 malfunction upregulates the Wnt/β-catenin pathway that is crucial to brain development and is often dysregulated in neuropsychiatric disorders. Moreover, the genes TCF4, CNTNAP2 and RELN, which are implicated in the aetiology of ASDs and SZ, are also upregulated. In addition, the results suggest that CHD8 modulates DLX gene expression and may affect GABAergic interneuron development.

Molecular mechanisms underpinning the abnormal brain vascularization observed in patients with SZ could be recapitulated in vascularized organoids [103]. Vascularized organoids can be obtained by embedding brain organoid and endothelial cells together in Matrigel matrix. To further induce the growth of blood vessels, these organoids must be transferred into the cortex of immune-deficient mice. After transplantation, positive blood vessels are formed inside and inbetween rosettes within the centre of the organoid. Moreover, the blood vessels of the graft organoids connect to the host blood vessel system to enable blood flow into the organoids [104]. We suggest using this model for further research on SZ.

## 5. Conclusions

GWAS-guided investigation of in vitro models will advance our knowledge of the pathogenic molecular mechanisms of SZ. There are relatively few genetically engineered models of SZ, considered in comparison with those available for other common polygenic diseases. Post-GWAS in vitro studies of SZ represent a promising way to dissect the functional roles of disease-associated genetic variations. A wide range of processes in the brain could be reproduced and studied using cellular models (Table 1). The choice of the proper model should be based on its constructive validity to the process being distorted in SZ. For example, protein–protein and protein–nucleic acid interactions can be reproduced in cellular models such as neuroblastoma cell lines, while synaptic transmission can be reconstituted in iPSC-derived neurons. Misbalance in cellular composition and abnormal cellular migration and localization are observed in specific neural-specific structures, including neural rosettes and brain organoids. Reduced myelination and neuro-inflammation can be modelled in co-cultures of neurons and glial cells. Each model has advantages and limitations that will affect its suitability for a given study (Table 2). The challenge for the coming years is to establish a streamlined and reproducible procedure for obtaining genetically edited brain organoids. The development of organoids that reconstitute all brain structures and vascular supply (including the blood–brain barrier) will facilitate the search for effective and personalized SZ treatments.

## Figures and Tables

**Table 1:** In vitro models used to study cellular and molecular SZ-associated phenotypes.

	Cellular and Molecular SZ-Associated Phenotypes					
Model	Protein Synaptic Density (PSD)	Functional Connectivity	Neurite Outgrowth	Synaptic Pruning/ Neuroinflammatory	Myelination	Neurodevelopmental (Tissue Microarchitecture, Differentiation, Proliferation)
Neuroblastoma (SH-SY5Y, SK-N-SH, SK-N-MS)	+ [16]	+ [105]	+ [106]	−	−	−
Neural progenitor cells (NPC)	−	+ [107]	−	−	−	+ [108,109]
iPSC-derived neurons (glutamatergic, dopaminergic, etc)	+ [110]	+ [111,112]	+ [113]	−	−	+ [14,39]
Co-culture models (neurons and glial cells)	+	+ [7]	+	+ [68,114]	+ [115]	+ [116]
Brain organoids	+	+	+	+	+	+

“+” denotes an obtained or potentially available model. “−” denotes a non-applicable model.

**Table 2:** Properties of in vitro models used in SZ research.

Model	Corresponding Cell Type or Process (Constructive Validity)	Advantages	Disadvantages	References
Neuroblastoma (SH-SY5Y, SK-N-SH, SK-N-MS)	Immature neurons	Low cost, ease of culture, convenient genetic manipulation, high reproducibility	Tumour origin and specific genomic alterations	[11,12,13,15,23,26,27,28,29,30,31]
iPSC/ESC-derived NPCs, glial cells and neurons	NPCs, glial cells and neurons	High similarity to native cerebral cell populations, patient’s genotype, various neuronal populations can be obtained depending on culture conditions	High cost, difficult culture conditions and genetic manipulations, mutational load and heterogeneity of source cells	[36,37,39,50,60,61,73,75]
iPSC/ESC-derived multicellular models (neural rosettes, neurospheres, glial and neural co-cultures)	Limited to processes of neural stem cell proliferation (neurospheres), neurulation (neural rosettes), differentiation and intercellular interactions (co-cultures).	Reconstitution of cellular interactions during early stages of brain development, lower cost compared to brain organoids	High cost, difficult culture conditions. Heterogeneity due to culture conditions, in addition to mutational load and heterogeneity of source cells. Limited validity, compared to brain organoids.	[84,86,87,88,90]
iPSC/ESC-derived brain organoids	Non-vascularized organoids—8–16 gestational week embryo brain, vascularized organoids—16–25 gestational week embryo brain	Native cerebral tissue architecture and signaling pathways preserved, cultivation conditions published for organoids of different brain areas	Highest cost, difficult culture conditions. Heterogeneity due to culture conditions, in addition to mutational load and heterogeneity of source cells.	[92,93,94,103,104]

## Data Availability

Not applicable.
